# Silence, Rebellion, and Acting-Out of a Silenced Past: Understanding the French Riots From a Postcolonial and Transcultural Perspective

**DOI:** 10.3389/fpsyt.2019.00909

**Published:** 2019-12-18

**Authors:** Malika Mansouri, Marion Feldman, Jonathan Lachal, Elisabetta Dozio, Mayssa’ El Husseini, Marie Rose Moro

**Affiliations:** ^1^ Université Paris Descartes, Laboratoire PCPP (Psychologie Clinique, Psychopathologie, Psychanalyse) EA 4056, Paris, France; ^2^ Université Paris Nanterre, Laboratoire EA 4430 CLIPSYD - A2P, Approches en Psychopathologie Psychanalytique, Nanterre, France; ^3^ CESP, Fac. de Médecine - Univ. Paris-Sud, Fac. de Médecine UVSQ, INSERM U1178, Université Paris-Saclay, Villejuif, France; ^4^ University of Picardie Jules Verne, CHSSC EA 4289, Maison de Solenn-Hôpital Cochin, Paris, France

**Keywords:** riots, urban poverty, colonialism, traumatism, adolescence, transgenerational

## Abstract

**Context and Objectives:** According to a sociological study, the adolescents involved in the “suburban riots” of November 2005 were French nationals with a foreign background, including 55% of North African descent. Numerous attempts to interpret the “riots” have been made, but none of them has discussed the impact of the “silenced” colonial history on their filiation. For this reason, the present research set out to overcome this shortfall.

**Methods:** Using a complementarist, transcultural, qualitative research methodology, 15 interviews with French adolescents of Algerian descent were analysed.

**Results, Analysis and Discussion:** The analysis of these interviews highlighted the impact of the past violence in France’s colonial history on family dynamics and intergenerational relationships, which seemed to play a crucial role in the unconscious component of transmission within these families. This discovery led us to a new understanding of the 2005 revolt, envisaging it as a symptom of a disorder situated on several levels: on the level of subjectivity, of trans-generational relationships, and also on the level of social cohesion within French society. The interviews showed how the young interviewees related their current anger to French colonial and post-colonial history.

**Conclusions:** These observations led to a new understanding of the “riots” as a form of acting-out of anger linked to contemporary and past experiences of domination and exclusion.

## Context

The November 2005 “suburban riots” in France led to a feeling of astonishment and bewilderment in French society. During a period of three weeks, these riots gave the police forces no rest. There were no official leaders and no particular demands, yet the riots spread across the whole French territory. They were sparked off by the death of Zyed Benna (17 years old) and Bouna Traoré (15 years old), two youngsters who died from electrocution while trying to escape from the police (1). What happened exactly? On October 27th, a group of about a dozen teenagers had been playing soccer. After play, they set off home for dinner (it all happened during the daytime hours of the Moslem Ramadan fast). As is often the case, the police were carrying out identity checks, focusing particularly on young people in the suburbs where there is a large proportion of families with a migrant background. What often happens when teenagers see police officers checking people’s IDs is that they run away. Zyed Benna, Bouna Traoré and one of their friends ran away from police officers who chased after them, even when they saw that the boys had gone into an electrical substation, trying to hide. All three of them sustained a powerful electric shock, which caused the deaths of Zyed Benna and Bouna Traoré. That same evening, violence broke out in their very poor eastern suburb in Paris. Initially confined to the Paris area, the unrest subsequently spread to other cities in France.

## Objectives

According to a sociological study (2, 3), the adolescents involved in these “suburban riots” were French nationals with a foreign background, including 55% of North African descent. The peculiarity of these rioters appears as their links with the history of France and its former ancient colonies. In a way, these young people may be considered as inheritors of the historical relationship between France and its former colonies. Although there have been many attempts to explain the “riots”, the impact of a “silenced” colonial history on these young people (including the impact on trans-generational relationships) has never been explored. For this reason, the present research set out to bridge this gap. We tried to understand whether or not the traumatic colonial past had an impact on their feelings, and consequently on the events, and how this came about. We also sought to understand what motivates French teenagers’ contemporary affiliations, and how they perceive their contemporary world.

## Methods

The study involved encounters with 15 young people of Algerian descent (proportionally the largest population of French nationals originating from former colonies) for individual research interviews, to understand the reasons for their anger. The 15 young people interviewed were young men aged 18 to 25 years (**Table 1**). The mean age was 20. Contacting these young people required anthropological work to be carried out in a context of cultural proximity, a sort of domestic anthropology in suburban Paris (4, 5). More precisely, it consisted in the researcher setting up residence in these districts for several weeks to facilitate encounters. Six interviews were conducted at the heart of the Seine-Saint-Denis urban estates with young people who were experiencing difficulties or who had dropped out of school. These interviews concerned Jawad, Aziz, Bilal, Farid, Amir and Mounir. One main issue was to find out whether this expression of anger could also be found among young people who were doing well at school. A recruitment of subjects from a university was thus decided, and over a two-year period, six further interviews were carried out. They involved Nadir, Younes, Karim, Habib, Adam, and Mourad. In addition, because these riots had spread across France, three young people were met outside the capital, through acquaintances: Tariq, Kamel and Salim. The interviews took place in a normal life setting between 2010 and 2011, in the aftermath of the “riots”, following a period of psychological elaboration during which memories were given form. They lasted between 30 and 90 min. The mean duration was 1 h. The subjects in this study were therefore teenagers at the time of the 2005 riots. The semi-directive interviews were focused on four lines of approach: the family’s migratory trajectory, to obtain a historical perspective; the perceptions of contemporary social reality; the perceptions of rebellion or rioting; and the representations linked to Franco-Algerian colonial history. The interviews were recorded and the verbatim was transcribed. All the participants gave their written informed consent. The young men could refuse to continue the interview at any time. For ethical reasons, the data shown here has been made anonymous.

**Table 1:** Socio-demographic data for the research population.

Name	Age	Studies/professional activity	Nationality	Parents’ origin
Jawad	20 years old	Dropped out of school at 16 Unemployed	French	Algerian
Aziz	22 years old	Cookery training course Works in a restaurant	French	Algerian
Bilal	20 years old	National Higher secondary Diploma	French	Algerian
Farid	19 years old	Dropped out of school at 16 Unemployed	French	Algerian
Amir	18 years old	Dropped out of school at 16 Is looking to further his education	French	Algerian
Mounir	25 years old	2 years post BAC in Law studies Community life officer	French	Algerian
Tarik	23 years old	3^rd^ year psychology student at university	French	Algerian
Kamel	25 years old	Technical diploma in mechanics Mechanic	French	Algerian
Salim	23 years old	BAC Unemployed	French	Algerian
Nadir	21 years old	1^st^ year at University for a two-year training qualification	French	Algerian
Younes	21 years old	1^st^ year at University for a two-year course	French	Algerian
Karim	18 years old	1^st^ year at University for a two-year course	French	Algerian
Habib	20 years old	1^st^ year at University for a two-year course	French	Algerian
Adam	20 years old	1^st^ year at University for a two-year course	French	Algerian
Mourad	18 years old	1^st^ year at University for a two-year training qualification	French	Algerian

The methodological approach applied to this transcultural research is based on Georges Devereux’s notion that human phenomena require the discourse from both psychology and sociology, but not simultaneously; indeed, a “raw fact” does not altogether belong to a specific domain. It is only through explanation within the field of either science that raw facts are converted into psychological or sociological data (6). This complementarist approach therefore requires the different theoretical fields of the social sciences to liaise, so as to facilitate the study of complex objects (7–9). If we consider that there are collective and historical components to humans’ subconscious singularity, our research contributes to a dialogue between history, psychoanalysis and post-colonial studies.

A longitudinal analysis was carried out, taking the form of individual portraits, followed by a cross-sectional analysis. This second step helped to evidence recurrent themes *via* the use of Grounded theory (10). This is an inductive research method aiming to build a theory based on the collection of empirical data. Unlike hypothetical and deductive approaches, the aim here is not to validate or invalidate an initial hypothesis. It is the data itself that shows the way. From this exploration, the material was interpreted, looking for links between what was said and what was not said, between discourse on past history and discourse on everyday life in the community. In a proximity with clinical practice, with all the complexity arising from the research perspective, we paid considerable attention to the unsaid, the allusions, the omissions, and what cannot be told and hence is not immediately obvious in the discourse of the different individuals. This data analysis method enabled the meaning of events to be highlighted, by linking a variety of elements within the same situation, whilst remaining centred on latent aspects of what the subjects expressed, particular attention being given to subjectivity and the search for explanations.

## Results and Analysis

### Institutional Relationships That Return to Patterns of Colonial History

The adolescents stated that the police did not treat them fairly, systematically suspecting any “Black” or “Arab” young person. According to Bilal, there is no need to have committed a criminal offence to be considered a suspect, merely walking in the street is enough to become one. According to Amir, policemen take all kinds of liberties towards them because “they know that even if we lodge a complaint against them, they will be the ones who win and not us, because we are from the suburban estates.” For them, encounters with the police during identity checks are the brutal expression of arbitrary force, and many of the youngsters linked this experience to the arbitrary segregation during the colonial period. The identity checks are experienced as an expression of inequality within French society. They nevertheless felt unable to contest this inequality, and many of them considered running away to be the only option they had when encountering police officers, even if they had not transgressed any law. Being confronted with the police is considered to be dangerous, even fatal, as in the case of the two youngsters who died in 2005. Some of the interviewees used the same terms to describe their encounters with the police as they used when talking about encounters their parents and grand-parents had had with colonial officials. They said: “the police harasses us every day” and “they oppress us.” And they used exactly the same words when they tried to explain how their parents lived during the colonial period. They said: “they harassed our parents” and “they oppressed them”.

School appears as a space where the desire to “get on” in the world is hampered by problems of segregation; it is a reminder of colonial times, when “indigenous” people were not expected to get far in their schooling (secondary school being generally the highest education reached). Tarik, who was in 3^rd^ year psychology at University, described suburban secondary schools as machines designed to “mis-(dis-)orient” “Black” or “Arab” young people by pushing them almost systematically towards short vocational training courses. That was what had happened to him. His teachers wanted him to enrol on a shorter training program. But Tarik’s elder brother, who had gone through the same process, opposed this decision. Some of the youngsters in this research said how bitter they felt about the fact that their parents had not been allowed to attend school or pursue an education in colonial and postcolonial times. Kamel asked: “My grandfather lived in poverty, my parents lived in poverty … Why was the opportunity to go to school not given to my mother?” Most of the youngsters described how they felt betrayed in their love for school when reaching the end of secondary school. With emotion, Younes remembered: “frankly, It was just like that when the time came to decide what course to choose, I used to cry a lot in the schoolyard*…*” Further on, he added: “we are not encouraged by teachers … We are discouraged to continue our schooling because they are convinced that “Black’ or “Arab’ learners cannot be successful beyond a certain level.” These young people felt a real sense of abandonment by the teachers.

Having inherited France’s colonial past, these youngsters experience the violence of a past racist phenomenon that seems to endure in today’s reality with its collective indifference. The absence of support in the youngsters’ failing environment is experienced as a structural disenchantment towards them. In this situation of deprivation (Hilflosigkeit), which has now been going on for several generations, they express overwhelming feelings of distress, hopelessness, worthlessness and helplessness.

### Identity

Most of the adolescents described the suburbs as a rough place where they are constantly confronted with exclusion and tempted to engage in delinquency. This was described as a real risk for the more vulnerable. A lot of them were jobless and felt left behind “because of what we are” and “what we are supposed to be,” as a result of their physical appearance. When talking about the suburbs, Karim said that “Black” or “Arab” families had been “parked” “on the fringes of the Republic” and that young people were born and grew up on these fringes. The birth of a child necessarily occurs within a specific lineage, with a place in the genealogy, history and geography. Each child has paternal and maternal affiliations, which later provide a sense of belonging to a community. If for most adolescents, puberty is experienced without particular difficulties, but for others, this period can be more difficult, and even stressful. From a dynamic perspective, puberty is characterized by a libidinal eruption. This energy leads adolescents to seek to discharge non-elaborated tension. With the reactivation of the Oedipus conflict, personal and sexual identities attempt to construct the Subject, while more archaic psychic conflicts weaken the Self, in its pare-excitation role. Furthermore, during adolescence, questions of filiation and affiliation take on specific meanings (5). Ontological, identity-related questions arise: Whom do I look like? To whom do I belong? And who do I want to be? (11). The psychological processes of adolescence run alongside changes in the body linked to puberty. It is a violent experience for adolescent subjects, a form of trauma that overwhelms them. This violence highlights the traumatic effect of puberty, “the occurrence of a psychological conflict and a traumatic development in pubescent adolescents” (12, p. 41). This violence is necessary for subjects who have lost their childhood status with its imaginary world and points of reference, only to be confronted with an extremely frightening newness. It is nevertheless necessary, since it allows new perspectives to be opened up towards individuation and subjectification. However, adolescents can also be tempted to rid themselves of this internal violence, which they express as a trauma when they are overwhelmed and not able to contain and transform this energy of violence (13). They therefore have no choice but to turn this violence against themselves or project it onto the outside world. This violence could be defined as a mode of defence against a real or imaginary attack, a weakness in their identities that makes them feel threatened. It is a psychological process of transformation that is consubstantial with becoming an adolescent. But the teenage years are harder for some than for others, because becoming a healthy functioning adult also depends on the social context. These young people experience their suburb as a space that is at once part of the French republic and not part of it. They described the strange experience of being, at the same time, inside and outside French territory. The experience is linked to shame, anger and disgust: one of the youngest interviewees, Habib, talked about the suburb being a “mirror” the young people were living with, a mirror to France’s colonial past, reflecting “a piece of shit that they have to eat every day.” When talking about the way they are perceived by others, many of them talked about images they had to deal with, and they described them as images that were constantly reproduced within French society. They are French, but at the same time they are treated as if they were not really part of French society. This recalls the second-grade status colonial subjects had in the past: the “indigènes” or “natives.” The Franco-Algerian philosopher Sidi Mohammed Barkat (14) described this “legal limbo” as neither real inclusion, nor full-blown exclusion, but the indefinite hanging on for some future inclusion. He argued that this legal limbo enabled the French to treat the colonized as a less-than-human *mass*, but still subjected to a humanizing mission; they were only able to become fully human once they had cast off all the features that the French used to define them as part of the “indigenous” mass. In a way, the teenagers saw themselves as being part of an image constructed by others: part of a de-individualized mass, being a “suburban youth,” a “delinquent,” or eternally a “child of immigration descent.” They considered that these labels identified them as undesired by society, in need of reform and discipline as though they were delinquents, thus misrepresenting their distress (15). Behind the rage they feel, these youngsters were ashamed of being seen as dangerous foreigners, or terrorists. Here, shame takes root in the feelings of being unworthy, being or having something that is not “normal” with regard to the social context.

### Fight for Life

There are many reasons for the anger that emerged in this revolt, but one thing that seemed important to us was that it was triggered by the *death* of the two adolescents. The young interviewees talked about them as victims of a “racist crime” reminding them of the day-to-day experiences of racism in their encounters with the police. A lot of them said “it could have been me or my little brother.” On a more intimate level, the death of these youngsters appears to mirror another deeper personal experience: the “death” experience of their subjectivity, the impossibility of being seen, or voicing their experiences. There are strong links between this experience and that described in relationship with the colonial past. We wonder whether these young people were not trying to force the dominant society to look at the silenced and “forgotten” components of the discriminatory past in French history and its impact on current French society—an intimate and collective rebellion that shows the will to exist, to struggle against depression and despair, and to fight the risk of implosion by externalising and exploding (16). In this adolescent revolt, the effects of collective denial alongside the parental traumas of the past, meet the potentially traumatic effects of “pubescent adolescents” (13). It is the articulation of all this violence that explains their violent acts, acts committed to remain visible. By rebelling against the established order that they consider unjust, these teenagers break its rules. Their violent acts could be understood as the only solution to the collective obstinacy, the violence, the deafness and the blindness, in a contemporary and historical context of abandonment. What the interviews showed were surprising parallels between the current experience of exclusion and the colonial past, even though these parallels were not elaborated. The anger, and sometimes the rage of these young people is linked to their everyday life experience of exclusion, but it also seems to mirror France’s colonial history. This is why the anger felt today is also linked to the non-elaborated cryptic transmission of a colonial past.

## Discussion

The adolescent interviewees described a collective amnesia concerning the colonial past, a subject that is never brought up or discussed in public spaces. This silencing prevents any understanding of the impact that this past has on the present situation or of the way these adolescents experience life in today’s world. The absence of elaboration of the colonial past in school, and the denial of the physical and symbolic violence of the colonial past, reinforces the family dynamics whereby traumatic experiences become unspoken “cryptic” elements thus triggering an acting-out of the violence that cannot otherwise be elaborated. As pointed out by Abraham and Torok, two psychoanalysts working on the transgenerational transmission of trauma, “cryptic phantoms” can persist and haunt generations, passing from parents to children. The phantoms seek resurrection, resuscitation, and this will occur even if children must silently incorporate them within their psyche. What Torok means by “phantom” is a formation in the dynamic unconscious that is found there not because of repression by the subject, but on account of a direct empathy with the unconscious or rejected psychic matter of a parental object (17). The violence of the past has given their parents a life of non-existence, which the children inherit in silence and experience for themselves. There are three fundamental aspects in filiation. The first one concerns the transmission of the family name, as the social norm defines it. It is linked to rules, laws, and institutions. The second one corresponds to the relationship between the mother and her child. The last one is related to narcissistic filiation, which concerns the affiliation process in its fantasised and imaginary dimensions (18). But for these adolescents, trauma has usually destroyed the first and the second aspects of the filiation link. We therefore consider these adolescents as having a traumatic filiation and affiliation. This effect adds complexity to their psychic construction: how is it possible for them to answer questions about their belonging? How is it possible to answer questions related to their identity? In the same way as their colonized parents, they seem to have only two possible choices, brought to light by Frantz Fanon (16): explosion, to protect themselves from implosion, or violence to protect themselves from petrifaction. In the “country of human rights” where we brag about the French model of integration, the young generation of North-African or other colonial descent is still feeling left behind by their own country, unfairly subjected to police checks, segregated in the low-class suburbs that are becoming ghettos, left to fail in school, jobless, etc. They are hopeless and think that the situation cannot change because they are not wanted by their country: France. Colonization is part of French history and if we want to avoid the acting-out of the unsaid, we should accept and discuss this past. France has to face its colonial past with its inheritance. The fourth generation of the so-called “children of migrants” is already here. They are also “our children of tomorrow” (5), that is why we need a “decolonization of the French imagination” (19). Then this French youth could finally constitute itself as a subject of memory and history, with the possibility of symbolizing, and remembering. This may be a way out of the “cul-de-sac” which appears as a central point in our research: a conflict-ridden hybridization. To restore the social link, we need to make sure that there is transmission and critical discussion of the past. The elaboration of history should be actively undertaken in social and political life, but it also should have its place in psychotherapy, especially in the context of trauma. As xxxx has said, we can see how the cultural, political and social parameters, in other words the collective parameters, complicate individual analyses, and how the way we look at these young people shapes them and sometimes imprisons them (5). We should think about our psychological conceptions of these teenagers’ problems and consider the impact of history on their experience.

Evidence for a close link with colonial history has been upheld in a book entitled *The French Intifada: the long war between France and its Arabs* (20) by Andrew Hussey, a British historian and a specialist in French culture. This “long war” can be seen in the unfolding of many contemporary violent debates on Islam and the wearing of the veil in France. The bodies of women have become a central issue in a fierce debate as a result of an unresolved colonial conflict. In this explosive context, citizens are starting to gather against the spread of islamophobia. It can also be added that Amnesty International, in a report on discrimination towards Moslems in Europe, published on April 24^th^ 2019, denounces French islamophobia.

## Conclusion

In a similar way to that described by Schneider (21) in the United States, in France minorities derived from the former colonies start riots because they are reduced to a sense of powerlessness, with no alternative, and because they have to face discriminatory police violence that is sometimes fatal and remains unpunished. This was also the case in the United Kingdom, particularly with the death of a young black man called Mark Duggan who was killed by the police in August 2011 in Tottenham. The judges concluded that the police were within their rights (22). In his book entitled “Why I am no longer talking to white people about race” (2017), Eddo-Lodge documented racial riots and confrontations involving fatalities in the United Kingdom from the 1960s up to the present. The fear, injustice and powerlessness felt by minorities faced with the risk of dying at the hands of the police is a theme common to all these riots. But our research brings to light an understanding of these phenomena that cannot be reached without exploring a past haunted by a historical disaster. It would be interesting to confront our research findings with other experiences, such as the riots in London and its suburbs. It would help to find other dimensions of the processes of silencing the past. We know that the rioters in London were essentially young blacks from poor neighbourhoods. What is their family background? What is the historical relationship between their origins and Britain? Even if the collective histories are specific and therefore different (slavery, colonialism, etc.), we know that cryptic phantoms can persist and haunt through generations, passing from parents to children. While this involves the descendants of colonised peoples, it can be wondered how this articulates among the descendants of the colonisers entertaining islamophobic positions today.

## Data Availability Statement

The datasets generated for this study are available on request to the corresponding author.

## Ethics Statement

Ethical review and approval was not required for the study on human participants in accordance with the local legislation and institutional requirements. The patients/participants provided their written informed consent to participate in this study.

## Author Contributions

MM: Researcher. MRM: Director of research. MF and ME: Helped with the transcription of the verbatim. ED and JL: Helped with the methodology.

## Conflict of Interest

The authors declare that the research was conducted in the absence of any commercial or financial relationships that could be construed as a potential conflict of interest.

The reviewer SO declared a shared affiliation, with no relevant collaboration, with several of the authors ME and MM to the handling editor.
